# The tethered flight technique as a tool for studying life‐history strategies associated with migration in insects

**DOI:** 10.1111/een.12521

**Published:** 2018-04-14

**Authors:** Melissa Minter, Aislinn Pearson, Ka S. Lim, Kenneth Wilson, Jason W. Chapman, Christopher M. Jones

**Affiliations:** ^1^ Department of Biology University of York, Heslington Way York U.K.; ^2^ Biointeractions and Crop Protection, Rothamsted Research Hertfordshire U.K.; ^3^ Computational and Analytical Sciences, Rothamsted Research Hertfordshire U.K.; ^4^ Lancaster Environment Centre Lancaster University Lancaster U.K.; ^5^ Centre for Ecology and Conservation University of Exeter Cornwall U.K.; ^6^ Vector Biology, Liverpool School of Tropical Medicine Liverpool U.K.

**Keywords:** Animal orientation, dispersal, insect movement, migration, tethered flight

## Abstract

1. Every year billions of insects engage in long‐distance, seasonal mass migrations which have major consequences for agriculture, ecosystem services and insect‐vectored diseases. Tracking this movement in the field is difficult, with mass migrations often occurring at high altitudes and over large spatial scales.

2. As such, tethered flight provides a valuable tool for studying the flight behaviour of insects, giving insights into flight propensity (e.g. distance, duration and velocity) and orientation under controlled laboratory settings. By experimentally manipulating a variety of environmental and physiological traits, numerous studies have used this technology to study the flight behaviour of migratory insects ranging in size from aphids to butterflies. Advances in functional genomics promise to extend this to the identification of genetic factors associated with flight. Tethered flight techniques have been used to study migratory flight characteristics in insects for more than 50 years, but have never been reviewed.

3. This study summarises the key findings of this technology, which has been employed in studies of species from six Orders. By providing detailed descriptions of the tethered flight systems, the present study also aims to further the understanding of how tethered flight studies support field observations, the situations under which the technology is useful and how it might be used in future studies.

4. The aim is to contextualise the available tethered flight studies within the broader knowledge of insect migration and to describe the significant contribution these systems have made to the literature.

## Introduction

An enormous number of insects undergo long‐distance aerial migrations to escape deteriorating habitats and seek more suitable environments. Using the Kennedy/Dingle definition of migration, these movements are defined as ‘persistent, straightened out movement, undistracted by cues that would otherwise halt such movements’ (Dingle, 2014). From an ecological and practical perspective, migratory journeys have profound implications for the spread of pathogens, the invasion of major agricultural and human health pests, the seasonal cycling of large quantities of biomass and the distribution of insecticide resistance alleles (Altizer *et al*., 2011; Dingle, 2014; Chapman *et al*., 2015; Hu *et al*., 2016). There is, therefore, considerable interest in accurately quantifying flight behaviour and capacity across a range of economically beneficial and non‐beneficial migratory insects.

Tracking animals as they migrate is technically challenging. In birds, mammals and fish, it is possible to use radio telemetry, GPS tagging and satellite data to observe movement over large spatial scales and track individuals throughout their journeys. In insects, their small size and general lack of specific return‐migration sites present a different set of challenges (Chapman *et al*., 2015). Current methods used for tracking and describing the patterns of insect migration over continental scales include entomological radar (Chapman *et al*., 2011; Hu *et al*., 2016), weather radar (Stepanian *et al*., 2016), stable isotopes (Miller *et al*., 2011), neutral genetic markers (Troast *et al*., 2016) and, more recently, online‐based citizen science projects (Stefanescu *et al*., 2013). The combined effect of these studies has greatly improved our knowledge of migration at the biogeographical level but gives little information about the movement patterns of individuals. They also cannot answer specific questions related to migratory physiology, behaviour and genetics, which require detailed investigation under controlled settings.

Laboratory‐based flight systems provide an invaluable tool for studying behaviour in insects that are difficult to observe or track without specialised and expensive technology (e.g. entomological radar; Chapman *et al*., 2011). They also provide a unique means of manipulating physiological and environmental factors and quantifying their effect on flight behaviour in controlled settings. Multiple systems exist for doing this, including wind tunnels (Vogel, 1966) (reviewed for the use in birds in Hedenström & Lindström, 2017) and free‐flight chambers (Byrne, 1999; Blackmer *et al*., 2004; Perez‐Mendoza *et al*., 2011). Recently, however, there has been a resurgence in the use of tethered flight systems as a means of determining the flight potential of migratory insects (Attisano *et al*., 2015; Jones *et al*., 2015; Cheng *et al*., 2016; Jones *et al*., 2016; Marti‐Campoy *et al*., 2016). Tethered flight mills enable the continuous measurement of flight parameters over a prolonged period of time and have been used for the past 50–60 years to study behaviour both during foraging flights [e.g. Honey bees (*Apis*) (Wells *et al*., 2016)] and during migratory and dispersal flights [e.g. Diptera (Hao *et al*., 2013), Coleoptera (Lopez *et al*., 2014; Hoddle *et al*., 2015), Lepidoptera (Colvin & Gatehouse, 1993b; Kong *et al*., 2010), Hymenoptera (Vogt *et al*., 2000; Wanner *et al*., 2006; Bruzzone *et al*., 2009), Orthoptera (Krogh & Weis‐Fogh, 1952; Kent & Rankin, 2001)]. Despite the extensive number of studies that use these techniques, this literature has never been reviewed or contextualised in relation to what is known from field observations. To address this, we provide a summary of the two primary tethered flight techniques: rotational flight mills and flight simulators. For each system, we give an outline of the available technology, including the technical limitations and field relevance of these methods, and describe its applicability to the understanding of migration in insects. In addition, we describe the contribution these techniques have made to our understanding of the ‘migration complex’, a suite of co‐adapted traits which are largely absent in non‐migratory species, and compare studies related to environmental, physiological and genetic aspects of migration. Our review focuses primarily on studies investigating migratory species (Dingle, 2014), although we also include findings related more closely to ‘dispersal’ (Clobert *et al*., 2001) in order to highlight key points.

## Tethered flight techniques

The vast majority of studies using tethered flight are performed in artificial conditions with reduced optical and sensory environmental stimuli and in experimental chambers that can only mimic natural ambient temperatures and lighting as best as possible. Furthermore, the flight of an insect must be free from any influence by wind. Many migratory insect species use the wind as a cue to initiate migratory flight, to facilitate lift, to maximise the distance travelled, or as a guide for correct orientation, or all of the above (Chapman *et al*., 2008; Hu *et al*., 2016). In combination with the weight and friction imposed by the tethering mechanisms, this leads to the commonly asked question: ‘Do tethered flight mills capture migratory behaviour as we would expect to observe in the wild?’ The answer to this is relatively simple: the observed behaviour is almost certainly not natural. The question then becomes: ‘How *similar* is this behaviour to what we might expect to observe in the wild?’. This has applied significance, as output parameter estimates are very useful for forecasting models (Wang *et al*., 2017) and give us insights we cannot gain from more traditional field studies, particularly as these experiments can be carried out under controlled experimental conditions. In this section, we outline the two most commonly used tethered flight techniques and review them from a technical perspective.

### Rotational flight mills

**Figure 1:**
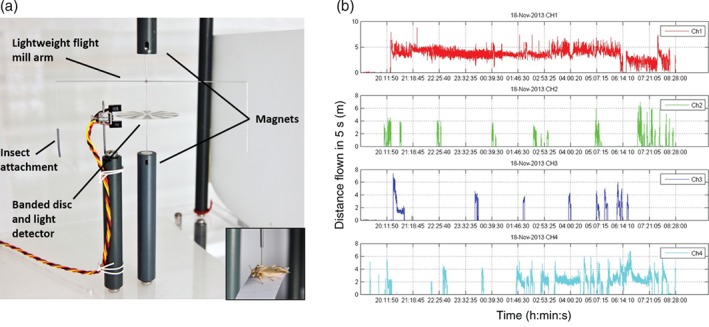
The rotational tethered flight mill system. (a) A diagram of a computerised tethered flight mill designed at Rothamsted Research (Lim et al., 2013). A lightweight flight mill arm is positioned between two magnets to reduce friction and encourage natural flight. The axis of the flight mill arm contains a striped disc which is scanned by the light detector on rotation of the mill arm. The light detector is wired to a computer connection in which the data are collected. The distance travelled is recorded to the nearest 10 cm and is updated every 5 s. The current system can fly 48 insects simultaneously. Insects are connected to one side of the mill arm via a pin attached to the thorax and fly in a circular trajectory with a one‐circuit circumference of 50 cm. (b) A time series plot of the flight activity of four adult moths Helicoverpa armigera flown over the course of the same night on the rotational flight mills between 19.00 and 08.30 hours (the following morning). [Colour figure can be viewed at http://wileyonlinelibrary.com].

Early designs of tethered flight go back as far as the 1950s with, for example, simple ‘roundabout’ mills designed to monitor the flight of up to 32 locusts simultaneously (Krogh & Weis‐Fogh, 1952). Another pioneering application of this technology was a set of experiments performed by Dingle on the large milkweed bug, *Oncopeltus fasciatus* (Dingle, 1965, 1966). The milkweed bug experiments relied on simple static tethered flight in which the insect is placed in a stationary position and the duration of wing beating is observed with individuals capable of a maximum mean total flight time of up to 12 h (Table 1). Similar approaches were used to characterise flight in other species (Padgham, 1983). In contrast, rotational tethered flight mills provide some simulation of mechanical air flow and visual cues (Cooter & Armes, 1993) and have been developed to electronically record semi‐free flight with many design iterations (see (Gatehouse & Hackett, 1980; Cooter & Armes, 1993; Lim *et al*., 2013;Jones *et al*., 2016 ; Marti‐Campoy *et al*., 2016) for examples). Although these custom designs possess slight variations, all are based on approximately the same principle: the insect is attached to one arm of a steel or plastic mill using a suitable adhesive, often via the back of the thorax, allowing the insect to fly in a continuous circle. The steel central axle is held vertically in place by the opposing repellent force of two magnets and can therefore turn with minimum friction once an insect initiates enough force to turn the arm (Fig. 1a). Data are recorded as the arm or an attached banded disc passes through an infrared or light sensor with flight speed reflecting the number of revolutions per unit of time. The time‐series data (Fig. 1b) can be converted into flight variables, including the distance, duration and speed of discrete individual flights, or combined as a summary to give the mean and total sum of flight variables for the total course of the experiment. Rotational flight mills are highly adaptable and more recent designs can accommodate insects of various sizes (ranging from 10 to 40 mm forewing length, and ∼10–1000 mg mass) (Jones *et al*., 2016; Marti‐Campoy *et al*., 2016).

**Table 1:** The maximum or mean flight duration of a selection of insects observed in experiments using rotational flight mills

Insect	Factor investigated†	Value taken	Maximum time flying (approx. hours)	References
Milkweed bug, *Oncopeltus fasciatus*	**Age**, **sex**, **mated status**	Maximum mean total flight duration	12 h	Dingle (1966)
Grasshopper, *Melanoplus sanguinipes*	**Lipid metabolism**, morphology, size	Maximum total flight duration	7 h	Kent & Rankin (2001)
Red palm weevil, *Rhynchophorus ferrugineus*	Sex, age, morphology	Maximum total flight duration	4 h	Avalos *et al*. (2014)
Cabbage webworm, *Hellula undalis*	Sex, age	Mean total flight duration	12 h	Shirai & Yano (1994)
Soybean aphid, *Aphis glycines*	**Age**, **temperature**, **humidity**	Maximum total flight duration	8 h	Zhang *et al*. (2008)
True armyworm, *Pseudaletia unipuncta*	**Sex**, **age**	Maximum total flight duration	10 h	Luo *et al*. (2002)
Cotton bollworm, *Helicoverpa armigera*	**Mated status**, sex, **reproduction/age**,**population**	Maximum total flight duration	10 h	Colvin & Gatehouse (1993b)
Black cutworm, *Agrotis ipsilon*	**Lipid/carbohydrate metabolism**	Maximum total flight duration	13 h	Sappington *et al*. (1995)
Desert locust, *Schistocerca gregaria*	**Metabolic rate, lipid/carbohydrate metabolism**	Maximum total flight duration	9 h	Weis‐Fogh (1952)

† Factors in bold typeface shown to affect flight duration in rotational flight mill experiments.

Tethering insects for flight provides a few technical challenges that must be considered prior to interpreting the output data. For the vast majority of distances flown by insect species on the flight mills, it is not possible to monitor the flight paths of unhindered individual insects in the wild and so some consideration must be given to how the act of tethering influences flight activity. A major shortcoming of the tethering technique is that insects do not need to generate the equivalent lift they would otherwise need to produce in free flight. A tethered insect does not support its own body mass and this may lead to unnatural flight behaviour and inaccurate reflections of natural flight performance (Dudley & Ellington, 1990; Riley *et al*., 1997; Snelling *et al*., 2012). This has been highlighted in locusts in which tethering results in lower flight speeds, wing beat frequencies and lift values equivalent to only ∼70% of their weight (Gewecke, 1975; Kutsch & Gewecke, 1979; Baker *et al*., 1981; Kutsch & Stevenson, 1981). For example, Baker *et al*. (1981) reported that free‐flying swarming locusts have higher wing beat frequencies (mean 22.9 Hz) and flight speeds (4.6 m s^–1^) than those in the laboratory (mean wing beat frequency, 19.8 Hz; mean flight speed, 3.3 m s^–1^). A way to overcome this is to use measurements of flight kinematics to monitor effort during tethered flight experiments (Snelling *et al*., 2012; Snelling *et al*., 2017). Secondly, the insect must produce enough force to turn the arm of the mill. If this weight is too great then the insect may forgo initial flight or, once in motion, may cease flying before the natural inclination to do so. For example, it was estimated that *Cicadulina* leafhoppers required at least 20–30% more energy to overcome the friction to ‘push’ the flight mill arm, and that the flight speed generated on the mills is about a third of what they would attain in free flight (Riley *et al*., 1997). This idea is supported by Taylor *et al*. (2010), who compared the flight performance of the emerald ash borer, *Agrilus planipennis*, on a flight mill and in free flight, and found that the free‐flight speeds were approximately three times greater than the speeds recorded by the flight mill. This means that maximum flight durations and speeds recorded on flight mills may underestimate the potential durations and speeds that are energetically possible. Lightweight arms with little friction have been designed to minimise this, allowing even relatively weak flyers to turn the mill (Jones *et al*., 2016). By contrast, the lack of opportunity for tarsal contact may overestimate flight potential. In the majority of rotational flight mill designs, tethered insects are not free to control their natural take‐off and landing. This means that insects may continue to fly in the absence of tarsal contact between flights (Edwards, 2006), overestimating the distances that may be flown in natural free flight. Flight mill systems have in fact been designed that incorporate a landing platform, and such a system has shown the tethered activity of the noctuid moth *Helicoverpa armigera* mimics that of field observations (active at dusk, settled by dawn) (Cooter & Armes, 1993), but this system has seldom been used in further experiments.

### How can rotational flight mill data be used to infer migratory behaviour?

An additional issue related to the flight mills is how to analyse the output data. Flight mills provide multiple measurements that can be used to interpret flight propensity. These are primarily speed, distance, duration or the number of individual flight bouts but also include characteristics such as the duration of the first or longest flight which, in relation to an individual's migratory tendency, may be more relevant. How to interpret all these variables in terms of flight performance has been dealt with differently across individual studies. The most common practice is to select an arbitrary cut‐off for one or two individual variables but this is likely to miss crucial information on different attributes related to migratory flight. For example, the timing of take‐off or the longest single flight may be more informative for certain migratory species. A full analysis of all output variables and their relative contribution to the overall variation in flight performance should be measured prior to any *post hoc* analyses. Finally, migration is a complex syndrome consisting of many behavioural, morphological and physiological traits. The very act of flying itself will be influenced by many of these traits, either directly or indirectly. Analyses of flight activity should therefore be integrated and modelled with as many measurable components that may co‐vary and influence performance on the flight mill. These can include simple morphological measurements (e.g. wing size, body mass) and developmental rates, or, if possible, flight fuel levels (e.g. lipids) and the expression of specific candidate genes for flight.

**Figure 2:**
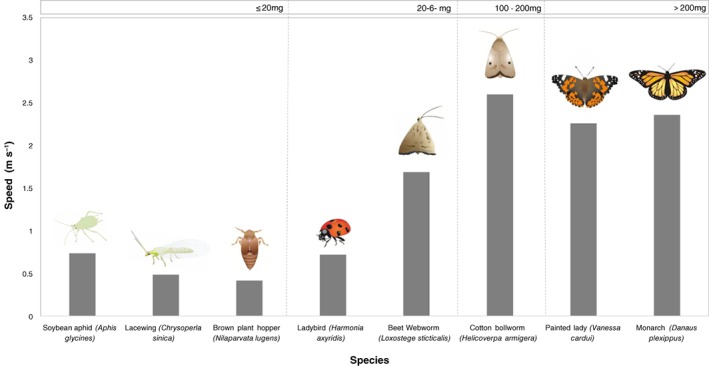
Recorded maximum speeds obtained by various species in studies using rotational flight mills. The maximum flight speed was taken for the soybean aphid, Aphis glycines (Zhang et al., 2008), green lacewing, Chrysoperla sinica (Liu et al., 2011), brown planthopper, Nilaparvata lugens (Zhao et al., 2011), harlequin ladybird, Harmonia axyridis (Lombaert et al., 2014), beet webworm, Loxostege sticticalis (Cheng et al., 2012), cotton bollworm, Helicoverpa armigera (Jones et al. unpublished data), monarch butterfly, Danaus plexippus (Davis et al., 2012) and the painted lady, Vanessa cardui (Jones & Minter unpublished data). [Colour figure can be viewed at http://wileyonlinelibrary.com].

The majority of research papers involving rotational flight mills aim to address the flight capacity of an insect during a predetermined length of time under experimentally manipulated conditions. This is usually addressed using insects caught from a wild population, reared under identical laboratory conditions and the flight performance of the adult progeny measured in response to a specific experimental treatment or in relation to a physiological parameter of interest. How far an insect can sustain flight depends on a myriad experimental and biological factors, but, when flown alongside conspecifics, the distance or duration of flight can provide a relative indicator of flight performance. Some individuals may readily undertake prolonged periods of continuous flight when attached to flight mills (Table 1). Figure 2 shows an example of the average speeds maintained by a range of species flown on flight mills. Adult moths of the beet webworm, *Loxostege sticticalis*, and cotton bollworm, *Helicoverpa armigera*, for example, have been observed to cover approximately 40–50 km over the course of a single night (Cheng *et al*., 2012; Jones *et al*., 2015). In contrast, other insects in the same experiment may engage in many short bouts (termed appetitive flights) or have little inclination to fly at all even if they come from populations containing long‐distance flyers. This raises the question of how ‘true’ migrants may be reliably distinguished from non‐migrants.

**Figure 3:**
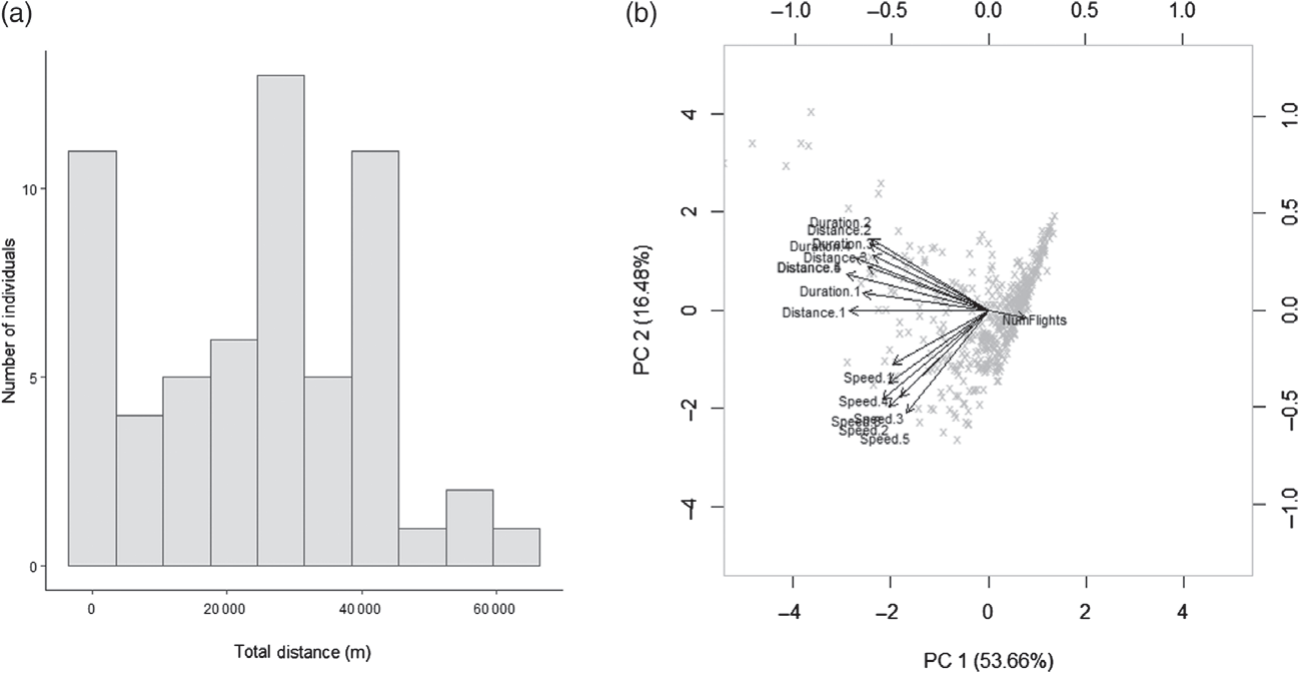
Variation in flight activity by Helicoverpa armigera recorded on the Rothamsted flight mill. (a) The total distance (m) covered by adult moths of H. armigera over the course of a single experiment. (b) Principal components analysis biplot of 16 tethered flight mill variables from flight activity of UK moths. The two first principal components (PC 1, PC 2) are plotted with the proportion of variance explained by each component printed next to the axis label, which together explain > 70% of variation in the data. Crosses indicate the 456 male individuals in the dataset; the top and right axes show principal component scores of the individuals. The arrows indicate the principal component loadings of the different tethered flight variables.

Following the Kennedy/Dingle definition of migration (Dingle & Drake, 2007; Dingle, 2014), it is anticipated that uninterrupted and sustained bouts of flight indicate a possible migratory response, especially in insect species that are known to be highly migratory within the pre‐reproductive period (PRP) (Johnson, 1969; Wilson, 1993; Colvin & Gatehouse, 1993a). Defining the criteria for classifying migratory insects is, however, open to interpretation. Many studies select arbitrary cut‐off values based on the duration or distance flown to assign individuals as long/short flyers or migratory/non‐migratory (Dingle, 1966; Rankin, 1980; Rankin & Rankin, 1980; McAnelly & Rankin, 1986a; Kent *et al*., 1997; Kent & Rankin, 2001). In the case of the North American migratory grasshopper, *Melanoplus sanguinipes*, flight duration is bimodal, with individuals tending to fly for less than 30 min or persistently for several hours (Kent & Rankin, 2001). This led to the adoption of the ‘1 hour’ rule in this species to classify grasshoppers as migrants or non‐migrants (McAnelly & Rankin, 1986a), which is highly repeatable (Kent & Rankin, 2001). In contrast, for many species the distribution of flight propensity is continuous and is, more often than not, negatively skewed, with a smaller fraction of insects engaging in longer/further flights (e.g. Schumacher *et al*., 1997b; Roff & Fairbairn, 2007). For example, Fig. 3a shows the distribution of the total distance flown by individuals of the migratory noctuid moth, *H. armigera*, during the course of a single night. Faced with this continuous variation in flight performance it is difficult to define an individual as truly migratory without selecting some arbitrary cut‐off value or outlier tail distribution. More detailed analyses beyond simple end‐point flight variables are therefore required to decipher potential migratory and non‐migratory behaviours from flight mill data. One example is from a recent study assessing the flight performance of several wild‐caught UK moth species representing insects of many different sizes and types (Jones *et al*., 2016). Rather than simply assess a single parameter, the authors performed a principal component analysis of 16 end‐point flight mill variables, and showed that, for the range of moths studied, total distance flown overnight and maximum flight speed were the most informative and captured most of the variation in flight (Jones *et al*., 2016) (Fig. 3b; unpublished data from *H. armigera*). In another example, to capture individual behaviour throughout the course of a flight experiment, a time‐series analysis was applied based on wavelets to describe changes in the periodicity of flights with regard to dispersal by woodwasps, *Sirex noctilio* (Bruzzone *et al*., 2009). By doing so, individuals were classified into ‘regular’, ‘periodical’ or ‘pulsating’ fliers and the flight patterns were strongly correlated with individual body mass. This novel approach could be extended to determine migratory from non‐migratory flight patterns.

### Flight simulators

**Figure 4:**
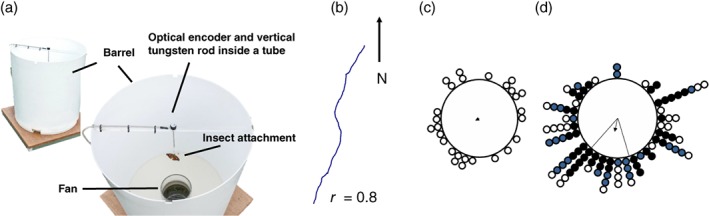
The flight simulator for determining flight orientation in day‐flying insects. (a) Diagram of the flight simulator designed by Mouritsen & Frost (2002). The insect is connected to an optical encoder via an attached pin on the back of the thorax that fits into a piece of plastic tubing at the end of a tungsten rod. The encoder possesses a low friction bearing allowing the insect to turn in any direction which is recorded to the nearest 3° every 200 ms. The fan at the bottom of the simulator creates a parallel air flow underneath the flying insect. (b) An example flight path produced by a painted lady butterfly, Vanessa cardui (Nesbit, 2009). (c) Individual mean flight headings of painted ladies flown under simulated overcast conditions; (d) individuals flown under clear‐sky conditions with visual access to the position of the sun during the autumn migration period and showing a mean orientation due south (Nesbit et al., 2009). Black arrows represent the mean direction with the length equivalent to the r‐value. Data were collected in 2006 (black circles), 2007 (grey circles) and 2008 (white circles). Solid lines are 95% confidence intervals. [Colour figure can be viewed at http://wileyonlinelibrary.com].

The second experimental system that uses insect tethering to infer migratory behaviour is the ‘flight simulator’ designed by Mouritsen & Frost (2002). Unlike rotational flight mills, flight simulators measure the orientation of insects as they fly in a horizontal plane. In this setup, insects are tethered to a vertical tungsten rod linked to an optical encoder which continuously records the in‐flight heading of the insect. The simulator is surrounded by a white barrel to reduce visual bias and a pipe at the bottom provides an optimal flow of air to promote active flight (a full description is provided in Mouritsen & Frost, 2002) (Fig. 4a). The output is a virtual flight path (Fig. 4b) that the individual takes during the simulation with a mean heading, which is advantageous as the entire track can be analysed for the preferred direction and the directedness of the flight path (Fig. 4c,d).

In the first experiments using this system, the mean flight orientation of eastern North American monarch butterflies, *Danaus plexippus*, matched closely the expected migratory direction (i.e. south‐west in autumn migrants heading from eastern North America to Mexico) (Mouritsen & Frost, 2002). Several other experiments have corroborated the robustness of this method in both *D. plexippus* (Froy *et al*., 2003; Merlin *et al*., 2009; Reppert *et al*., 2010) and the painted lady butterfly, *Vanessa cardui* (Fig. 4c,d) (Nesbit *et al*., 2009). As such, flight simulators are considered a highly successful means of determining the heading of migratory flight in large day‐flying butterflies. Reliable and repeatable headings for nocturnal species have been more difficult to obtain (Nesbit, 2009) and an extension of this method to other insect groups would be highly beneficial going forward. This may require some alterations to current designs. The small amount of friction required to turn the tungsten rod is easily overcome by large migratory butterflies but may limit smaller insects; with alterations, this system could be adapted to understand compass orientation systems in smaller migratory species, including key taxa such as mosquitoes where much remains to be understood about their migratory behaviour (Dao *et al*., 2014). As with the rotational flight mills, the flight simulators have limitations, including lack of tarsal contact, and it is unknown how much energy is needed to ‘turn’ the tungsten rod. One of the major advantages of the flight simulator is that experiments can be performed relatively easily in the field (Mouritsen & Frost, 2002; Merlin *et al*., 2009; Nesbit *et al*., 2009). This improves exposure to natural environmental stimuli such as temperature and humidity. As visual interactions with the environment have been shown to have a role in controlling flight in insects (Fry *et al*., 2009), the use of an optical flow and/or cues inside the barrel offer additional means of investigating the factors that affect flight performance in migratory insects.

### Future approaches for interpreting tethered flight data in relation to field estimates

The greatest potential for understanding how results from tethered flight mill studies relate to field behaviour comes from combining experimental techniques. For example, comparing flight propensity of field‐caught insects with signatures from vertical‐looking radar has the potential to significantly advance our understanding of how variable flight performance is under different environmental conditions. This would also enable direct comparisons of flight speed – information that is highly valuable when developing flight projection models for applied purposes such as forecasting the movement of insect pests (Wang *et al*., 2017). One possible approach to distinguishing migratory from non‐migratory individuals in a mixed population is a comparative performance analysis of flight simulators versus the rotational flight mills. If individuals that demonstrate clear flight path headings are identified as true migrants [as has been demonstrated with the painted lady butterfly, for example (Nesbit *et al*., 2009)], then these same insects could be flown on the rotational tethered flight mills. As long as the insects are flown within the expected migratory time frame, and are not damaged between flight trials, then a comparison of the data could yield interesting insights into the relationship between orientation and flight propensity. This experiment is particularly attractive in facultative migrants that generally comprise populations containing a mixture of both migrants and non‐migrants. Combining these techniques may provide researchers with a method to conclusively distinguish migratory from non‐migratory insects and such data can be incorporated into modelling of insect movements. For example, Wang *et al*. (2017) included the number of consecutive days of flight and average flight speed from tethered flight experiments into a forward trajectory analysis to estimate landing areas and migration duration of the rice leaf folder, *Cnaphalocrocis medinalis*. A drawback with this approach is the reliability of using the ‘self‐powered flight speed’ of *C. medinalis* (0.8 m s^–1^) (Wang *et al*., 2017) from flight mill data given the technical limitations described earlier. The development of correction factors that can account for the discrepancy between tethered and free flight would be a welcome avenue of research.

A consequence of sub‐maximal flight performance due to the limitations in the tethered flight method is that metabolic rates of insects during tethered flight may not represent free‐flight energy costs (Snelling *et al*., 2012). A solution to this problem is to combine tethered flight performance with metabolic (oxygen expenditure) and kinematic (e.g. wing beat frequency, lift, thrust, wing stroke amplitude) outputs. These measurements can be compared in both tethered and free‐flight assessments to identify periods in which tethered flight efforts are representative of free‐flight values. Using a respirometry system to quantify maximum oxygen consumption during tethered flight, Snelling *et al*. (2012, 2017) showed that locusts undertaking tethered‐flight can elicit periods in which metabolic rate, lift, wing beat frequency and wing stroke amplitude are comparable to free‐flight values. This is in contrast to earlier estimates (Gewecke, 1975; Kutsch & Gewecke, 1979). The modest energetics and kinematics of flying locusts most likely reflect the relatively small‐sized flight motor and load‐lifting capacity of locusts (Snelling *et al*., 2012), compared with, say, bees and moths, for which tethered flight can result in significant suboptimal flight performance (Heinrich, 1971; Dudley & Ellington, 1990; Harrison & Fewell, 2002).

## The influence of environmental factors on tethered flight performance in migratory insects

Insects migrate to new breeding grounds as an adaptive response to changing conditions in the surrounding environment. The environmental triggers that can potentially induce migratory behaviour include photoperiod, temperature, crowding and host‐plant availability/quality. The transformation of a non‐migratory insect into a migratory individual or ‘morph’ may occur at key developmental decision points in the insect's life cycle, in a classic example of phenotypic plasticity (Brisson & Davis, 2016). Tethered flight mills have been used as a simple means to determine the relative contribution of environmental factors on migratory flight activity both during development and during the course of flight. Some specific examples of how different biotic cues can influence flight activity are given in the following sections.

### Temperature effects

**Figure 5:**
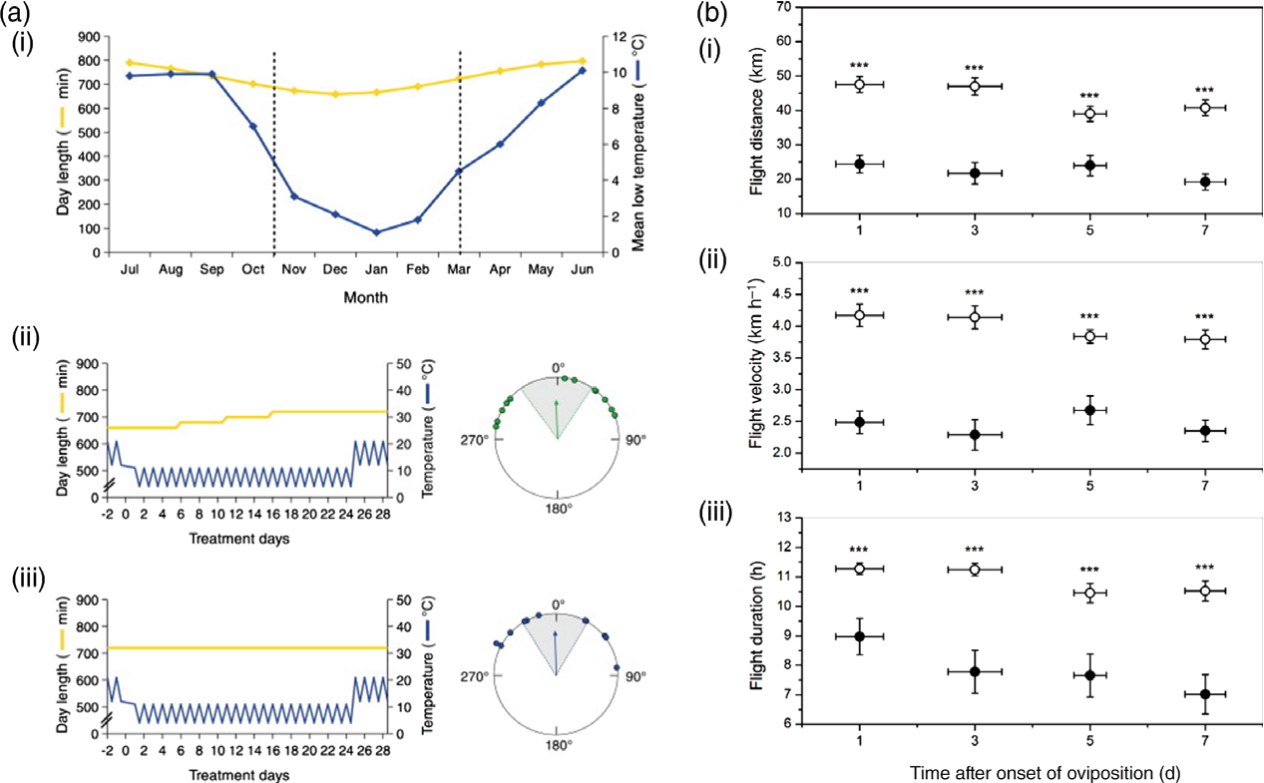
Insights into the migratory syndrome from tethered flight studies. (a) Coldness influences the direction of migratory flight in monarch butterflies, Danaus plexippus (Guerra & Reppert, 2013). (i) Mean monthly day lengths (yellow) and mean monthly low temperature (blue) at the monarch overwintering site; (ii) increasing photoperiod and low temperatures shift the orientation of fall migrants north; (iii) this orientation remains the same under constant photoperiod. (b) The association of oviposition and flight propensity in the female beet webworm, Loxostege sticticalis (Cheng et al., 2016). (i–iii) Changes in flight distance (m) (i), flight velocity (km h^–1^) (ii) and flight duration (h) (iii) with increasing days after oviposition with mated (solid circle •) and virgin (empty circle ○) females. [Colour figure can be viewed at http://wileyonlinelibrary.com].

Insects emerging at latitudes with average temperatures above or below the limits that can support their life cycle must either migrate or undergo seasonal diapause. It is not surprising, therefore, that optimum flight performance across several insect orders occurs at temperatures within their habitual range, with extreme hot or cold conditions inhibiting flight activity (Shirai, 1998; Lu *et al*., 2009; Jiang *et al*., 2011; Liu *et al*., 2011). While fewer studies have looked at the impact of temperature during development on flight performance, in one example with the Oriental armyworm, *Mythimna separata*, adult moths emerging from a strain reared at constant high temperatures flew significantly less than at optimum rearing conditions (Jiang *et al*., 2000; Jiang *et al*., 2003). In a clear example of how temperature during development can influence migratory flight, when autumn migrants of the monarch butterfly due to head south‐west were exposed to a premature coldness experienced at the overwintering site (11 °C light, 4 °C dark), butterflies shifted their orientation north when flown on the flight simulators (Guerra & Reppert, 2013) (Fig. 5a). This demonstrated that temperature in the microenvironment at the overwintering site is critical for calibrating insect orientation and for completion of the migratory cycle in this species.

### Photoperiod

The most reliable predictor of the changing seasons is day length, and many insects are thought to use photoperiod as a cue to engage in migration. Rotational flight mills have seldom been used to understand the impact of photoperiod. In the few published examples, there is little experimental evidence showing significant effects of decreasing/increasing shifts in daylight on flight activity (Jiang *et al*., 2011). So far, the best examples of experimental shifts in photoperiod disrupting migratory behaviour come from flight orientation studies. By phase‐shifting the clock of monarch butterflies, insects alter their orientation in agreement with the use of a time‐compensated sun‐compass (Mouritsen & Frost, 2002). Indeed, flight simulator trials have contributed greatly towards the elucidation of the sensory, neurocircuitry, molecular and physiological mechanisms of the sun‐compass in perhaps the best‐studied insect navigational system (Merlin *et al*., 2009; Merlin *et al*., 2012; Guerra & Reppert, 2013). It would be interesting to extend this work to see whether similar day‐flying migratory species, besides the Lepidoptera, use time compensation as a navigational tool and whether gradual changes in photoperiod, experienced during development, influence flight propensity on the flight mills.

### Density‐dependent effects

Locusts and aphids display classic cases of polyphenism in which the migratory or dispersive form develops under high‐density conditions (Simpson & Sword, 2008; Brisson, 2010). Density‐dependent migration is also, however, a key life‐history strategy in insects that do not possess morphologically distinct migratory morphs (e.g. wing monomorphic), and for these species tethered flight represents a useful tool with which to measure the flight response under crowded conditions. The impact of larval density on adult flight potential has mainly been studied in migrant Lepidoptera. Rearing larvae in crowded conditions increased the flight propensity of African armyworm, *Spodoptera exempta* (Parker & Gatehouse, 1985a; Woodrow *et al*., 1987), beet webworm, *Loxostege sticticalis* (Kong *et al*., 2010) and Oriental armyworm, *Mythimna separata* (Jiang *et al*., 2011), although no effect was observed with the black cutworm, *Agrotis ipsilon* (Sappington & Showers, 1992a). In the studies that show a positive effect between larval density and flight distance/duration, the relationship appears to be non‐linear, with a decrease in flight performance at the highest densities. This suggests that overcrowding increases physiological stress and reduces the resources available for long‐distance flight and that there may be a species‐specific density threshold.

### Insect–pathogen interactions

Migratory disease ecology, which is the study of pathogen evolution and disease dynamics in migratory populations, has been a subject area of intense research interest over the last 5–10 years (Altizer *et al*., 2011). Studies in this area have tended to focus on mammals and especially on birds, with relatively few insect studies. One exception is the monarch butterfly, *D. plexippus*, and its protozoan parasite, *Ophryocystis elektroscirrha*, which has been extensively used as a model system. The monarch has been shown to exhibit both migratory escape (healthy individuals escaping diseased regions; Altizer *et al*., 2000) and migratory culling (infection limiting the ability to migrate; Satterfield *et al*., 2013) at a population level. Tethered flight mills have proven valuable in elucidating the mechanisms behind these two phenomena, showing not only a reduction in the speed and duration of flight activity in parasitised butterflies but also proportionally higher levels of weight loss (Bradley & Altizer, 2005). Where this has been investigated in migratory insects, a reduction in flight capacity seems common (Seyoum *et al*., 1994; Dorhout *et al*., 2011). Tethered flight and its application in insect systems also allow us to investigate aspects of movement behaviour that would be technically challenging in larger animals. For example, this is one of the few experimental systems where flight effort can be manipulated, allowing us to study the relationship between disease tolerance and pathogen load while controlling for migratory effort (Chapman *et al*., 2015). Given the significant contribution of tethered flight studies to our understanding of the traits that make up the migratory syndrome, there is also huge potential to gain theoretical insights into how the two fields of migration and disease ecology relate to each other. Early work in this area, for example, suggests that disease may have higher costs in newly emerged adults, which invest the greatest effort in flight and are assumed to be in their PRP (see later) (Dorhout *et al*., 2008; Dorhout *et al*., 2011).

## Understanding migratory physiology using tethered flight

Migratory insects must make significant physiological adjustments to prepare for and initiate migration. In this sense, perhaps some of the largest contributions of tethered flight to the study of insect migration have been towards the understanding of reproduction–migration interactions and how insects fuel impressive long‐distance flights.

### Trade‐offs between migration and reproduction

In several migratory insects there is a trade‐off between the onset of migratory behaviour and reproduction; termed the ‘oogenesis‐flight’ syndrome (Rankin *et al*., 1986). The syndrome explains the pattern when egg development and mating are suppressed during migratory flight and subsequently stimulated after migration is terminated. Peak flight activity during pre‐reproduction as well as a decrease in flight performance with the onset of oviposition has been shown in many species as evidence of the oogenesis‐flight syndrome (Dingle, 1966; Armes & Cooter, 1991; Colvin & Gatehouse, 1993b; Schumacher *et al*., 1997b; Cheng *et al*., 2016) (Fig. 5b). The syndrome is not, however, universally present among all migratory insects, and tethered flight has been used to show that some species are capable of long‐distance flights both post‐mating and post‐oviposition (Sappington & Showers, 1992b; Schumacher *et al*., 1997b; X. F. Jiang *et al*., 2010). Additionally, long‐distance flight may accelerate the onset of egg maturation and oviposition (Slansky, 1980; McAnelly & Rankin, 1986b). Given the close relationship between reproduction and flight activity, it is not surprising that age is a significant factor when flying insects on flight mills and *a priori* knowledge of the species' biology is a necessity if using these assays to study migration. Indeed, age is the most common factor investigated using flight mills to determine the timing of peak flight activity as a proxy for characterising the migratory window in the field (e.g. Dingle, 1965; Armes & Cooter, 1991; Sappington & Showers, 1991; Liu *et al*., 2011; Lopez *et al*., 2014).

### Hormonal control of migration

Another key component of the migratory syndrome investigated using tethered flight is the role of juvenile hormone (JH). Levels of JH are intricately linked with migration‐reproduction resource allocation, with JH titres being lower pre‐reproduction and during the migratory window in those species that express the oogenesis‐flight syndrome (McNeil *et al*., 1995). Long‐distance flight activity itself stimulates the production of JH as a trigger for the onset of reproduction, and applications of JH or JH mimics have been shown to decrease flight activity on flight mills (Min *et al*., 2004a; Jiang *et al*., 2011). In contrast, in flight simulation studies using the monarch butterfly, topical application of the JH analogue methoprene failed to disrupt time‐compensated directionality, providing evidence that the maintenance of orientation is independent of JH levels (Zhu *et al*., 2009).

### Fuelling migratory flight

Insect flight is one of the most energetically demanding exercises in the animal kingdom and requires a suite of adaptations to ameliorate such a high metabolic demand (Arrese & Soulages, 2010; Snelling *et al*., 2012). Tethered flight has been instrumental in piecing together the metabolic timeline of long‐distance flights. In locusts, for example, for the first 10–15 min of flight, carbohydrate is the primary fuel source during which they fly at high speed (Goldsworthy *et al*., 1979). After this period, locusts slow down to a ‘cruising speed’ and the flight muscles switch to oxidising the higher‐energy content found in lipids with 1 mg of lipid creating as much energy as 8 mg of glycogen (Beenakkers *et al*., 1981). A number of flight mill studies have demonstrated that other insects also use lipids as the primary migratory fuel (Vanhandel, 1974; Teo *et al*., 1987; Sappington *et al*., 1995; Kent *et al*., 1997; Murata & Tojo, 2004). The switch to lipids from carbohydrates is mediated by adipokinetic hormones (AKHs) that mobilise stores of lipids (stored as triacylglycerol) from the fat body (reviewed in Goldsworthy & Joyce, 2001), and mean AKH titres are correlated with this switch during long‐distance flights performed by migrant individuals of the grasshopper, *M. sanguinipes* (Min *et al*., 2004b).

### Insect morphology and migratory flight

Flight performance is generally positively correlated with the body mass of the insect, largely reflecting the proportional increase in fuel load (Roff, 1991). Both body mass and wing length have been shown to predict flight activity in several flight mill studies (e.g. Gunn & Gatehouse, 1993; Attisano *et al*., 2013; Lopez *et al*., 2014). In a less obvious relationship between flight and a morphological characteristic, wing colour is associated with flight performance in monarch butterflies, with individuals presenting darker orange wings (approaching red) flying longer than those with a lighter orange pigment (Davis *et al*., 2012). This finding has been corroborated in field‐caught migrants (Davis, 2009) and suggests covariation between the deposition of wing pigmentation during metamorphosis and other life‐history traits that influence flight.

## Combining insect genomics and tethered flight for understanding the migration syndrome

The relatively short lives of insects mean that seasonal migration typically occurs over multiple successive generations. The migratory syndrome must, therefore, have a genetic basis and, in the case of facultative migrants, is likely to involve additional genotype–environment interactions (see earlier) and possibly epigenetic regulation, including the changes caused by modification of gene expression. Experimentally characterising reliable and robust migratory phenotypes is one of the biggest challenges in determining the genetic basis of migration (Liedvogel *et al*., 2011). Unlike larger migratory taxa (e.g. birds), insects are more amenable to the study of migration genetics as they can be reared in relatively large numbers under controlled conditions. Tethered flight represents a promising approach in which flight activity can be used as a proxy for determining migratory phenotypes for genetic and artificial selection studies. Determining these phenotypes can be complicated by the large amount of variation in migratory potential and flight activity both within and between populations of many migratory species.

Artificial selection experiments have shown that flight activity has a genetic component. In perhaps the clearest example of this, Kent and Rankin (2001) used the ‘1‐hour rule’ for the grasshopper *M. sanguinipes* (an insect is classed as a migrant if it makes at least one 60 min flight over several trials) to cross migrants and non‐migrants as well as to make reciprocal crosses between the two groups. Heritability (*h* ^2^) of flight activity (or migratory incidence) was estimated at 0.40. Similar estimates of heritability for flight duration have been reported in the noctuid moths *H. armigera* (*h* ^2^ = 0.39; Colvin & Gatehouse, 1993b), *Mythimna separata* (*h* ^2^ = 0.27; Han & Gatehouse, 1993) and *S. exempta* (*h* ^2^ = 0.40; Parker & Gatehouse, 1985b) as well as migratory moths from other families: *Cydia pomonella* (*h* ^2^ = 0.37–0.57; Schumacher *et al*., 1997a) and *Epiphyas postvittana* (*h* ^2^ = 0.43–0.57; Gu & Danthanarayana, 1992). These estimates are similar to those observed for migratory activity in other migratory taxa such as passerine birds (Berthold & Pulido, 1994).

While tethered flight has demonstrated that genetic variation in both flight propensity and orientation exists, the causative genes or variants (e.g. single nucleotide polymorphisms) have yet to be identified. One of the central questions is whether the phenotypic variance in migratory flight is due to a few genes of large effect or many genes of small effect. Migratory insects and tethered flight offer a robust experimental system for answering this question with several potential approaches. First, gene mapping (or quantitative trait locus mapping) has specific advantages in migratory insects over other larger migratory animals in that greater sample sizes can be generated to provide sufficient pedigrees from which genetic markers can be associated with the trait of interest. Constructing selected lines based on flight activity is still labour‐intensive; however, and a simple candidate gene approach may offer a less expensive and quicker initial alternative. For orientation in day‐flying insects, the molecular basis of the time‐compensated sun‐compass has been fully elucidated in the monarch butterfly (Merlin *et al*., 2009) and the role of the genetic components of this compass, including the light‐dependent cryptochrome genes, are well characterised in insects (Yuan *et al*., 2007). This offers an ideal set of candidate genes for understanding the genetic variation in orientation strategies in other species. With regard to flight propensity determined on the rotational flight mills, few examples linking genetic variation or gene expression (differences in transcripts of protein‐coding genes) to this trait exist. This is most likely due to both a lack of *a priori* information and the few available fully sequenced genomes of migratory insects (with exceptions; Zhan *et al*., 2011; Wang *et al*., 2014). This is all set to change with reduced costs for whole‐genome sequencing (WGS), as well as reduced representation *de novo* sequencing techniques such as RNA sequencing (RNA‐seq). A WGS population study of migratory and non‐migratory monarchs showed positive selection acting against a subunit of the flight muscle protein collagen‐IV (Zhan *et al*., 2014). However, for many migratory insects that possess discrete populations of migrants and non‐migrants, phenotyping assays are required for such comparative analyses. For example, this approach recently identified significant interpopulation differences in the flight performance of the cotton bollworm moth, *Helicoverpa armigera* (Jones *et al*., 2015). By classifying short‐ and long‐distance flight phenotypes, the authors applied an RNA‐seq approach to determine that flight performance was linked to the differential expression of a suite of candidate genes related to flight physiology, including hormonal control, flight muscle structure, odorant binding proteins and lipid metabolism (Jones *et al*., 2015). Differentially expressed genes from these studies can be used as candidate genes in other migratory species flown on tethered flight mills.

Ultimately, the overriding goal of laboratory flight genetic studies is to demonstrate a functional link between genotype and migratory phenotype. Tethered flight is perfectly suited as an *in vivo* phenotyping tool to test candidate genes in functional genomic assays. Progress has already been made in this area using genome editing technologies such as zinc‐finger nucleases, transcriptional activator‐like effector nucleases (TALENs) and CRISPR/Cas9 to introduce mutant components (e.g. *cryptochrome 2*) of the time‐compensated sun‐compass (Merlin *et al*., 2013; Markert *et al*., 2016). Disruption to the flight path headings of migratory monarchs possessing these edited genomes has yet to be tested, but the approaches described offer a useful template for researchers interested in functionally characterising migration‐associated genes, especially in non‐model organisms.

## Summary

Tethered flight represents a relatively simple laboratory assay with which to study traits associated with migration in insects. Here, we have attempted to summarise some of the applications of this technology to the field of insect migration. In a thorough literature search we identified 85 studies that have directly incorporated at least some aspect of tethered flight into experiments (Supplementary Table S1), reflecting the simplicity of the technique. Considering the limitations highlighted earlier, the conclusions drawn from tethered flight and translated to migratory behaviour in the wild should be treated with caution. For each species under study, efforts should be made to understand how factors such as the absence of tarsal contact or lack of natural lift required to support body mass influence flight outputs and how this relates to energetic expenditure of the insect. The biggest challenge still to be addressed is how flight patterns observed on rotational flight mills can be used to define true migratory from non‐migratory behaviour without relying on assumptions related to the biology of the insect. Improvements in this area are therefore urgently needed. Nevertheless, the strong correlation between the timing and duration of tethered flight activity with that of migrants in the field means that the technology holds much promise for elucidating new avenues of insect migration research. These include identifying the transcriptomic, genomic and epigenetic bases of migration (Liedvogel *et al*., 2011; Jones *et al*., 2015), mechanisms of navigation in nocturnal migrants (Warrant *et al*., 2016) and the relationship between migration and disease (Altizer *et al*., 2011). Integrating and interpreting the data from tethered flight mills alongside other techniques, such as entomological radar, field sampling, behavioural experiments and population genetics, will undoubtedly contribute to understanding the full migratory cycle of some of our most iconic and important migrant insects.

## Supporting information


**Table S1.** Identified studies which use the tethered flight technique to study flight and life‐history strategies in migrating insects.
